# Antimycobacterial peptides as natural therapeutics for tuberculosis: mechanisms and structural features

**DOI:** 10.3389/frabi.2026.1769369

**Published:** 2026-05-07

**Authors:** Pulak Pritam, Sreelipta Das, Sandeep Kumar Behera, Monali Sahoo, Lopamudra Subudhi, Shibani Mohapatra, Alok Kumar Panda

**Affiliations:** 1 Environmental Science Laboratory, School of Applied Sciences, Kalinga Institute of Industrial Technology, Deemed to be University, Bhubaneswar, India; 2 Centre for Biotechnology, Siksha O Anusandhan (Deemed to be University), Bhubaneswar, India; 3 Centre for Water Research and Climate Change, Kalinga Institute of Industrial Technology Deemed to Be University, Bhubaneswar, Odisha, India

**Keywords:** antimicrobial peptides, antimycobacterial peptides, mechanism, *Mycobacterium tuberculosis*, tuberculosis

## Abstract

The disease tuberculosis, caused by *Mycobacterium tuberculosis*, is one of the leading causes of global human mortality. The rise of multidrug and extensively drug-resistant strains of the pathogen and the limited efficacy of the BCG vaccine is one of the major concerns worldwide. Conventional chemotherapy for tuberculosis is often very long and has several side effects. These factors lead to an urgent need for alternative, non-toxic therapeutic strategies with minimal side effects. Antimycobacterial peptides (AMPs), are a class of natural compounds that have shown a broad spectrum of anti-mycobacterial activity with a low propensity for the development of anti-mycobacterial resistance. This review summarizes the current antimycobacterial peptides, highlighting their structural features, physicochemical determinants, and their mechanism of action. Some of the key peptides have been critically discussed with respect to their membrane targeting mechanism. The role of structural modifications, such as disulfide bonding, cyclization, hydrophobicity tuning, and post-translational modifications, in enhancing antimycobacterial efficacy and stability is also examined. Consequently, the broad mechanism of action of these peptides and their role in the development of anti-tuberculosis drugs have been emphasized. This article combines mechanistic and structural insights to show how antimycobacterial peptides could become new anti-TB drugs. It also provides a guide for developing and improving peptide therapies for tuberculosis.

## Introduction

*Mycobacterium tuberculosis*, the etiological agent causing tuberculosis, is one of the most common contagious diseases that poses a serious threat to human health. As per the recent WHO report, TB incidence throughout the world amounts to 10.7 million and the total death cases due to tuberculosis is around 1.23 million. In addition, the emergence of multidrug and extreme drug resistance TB which is around 390,000, adds to the additional burden of TB. The MDR/XDR tuberculosis is mainly due to the development of resistance by the pathogen against two powerful anti-TB drugs, i.e. isoniazid and rifampicin. The first line of anti-TB drugs, i.e. isoniazid, rifampicin, pyrazinamide, and ethambutol, are mostly effective against drug-sensitive TB but ineffective against the drug-resistant strains of *M. tuberculosis* (Bhagwat et al., 2022; Jones et al., 2022).

The pathogen *Mycobacterium tuberculosis* is one of the top fifteen causes of death worldwide and hampers the progress rate towards the target of the United Nations Sustainable Developmental Goal of Good Health and Well-being, i.e. SGD-3. The increasing prevalence of MDR (multidrug-resistant) and XDR (extensively drug-resistant) tuberculosis has further hampered disease control, and it enhances the economic burden on patients in low-income countries. This obstructs progress towards SDG 1 i.e. No Poverty, and SDG 10 i.e. Reduced Inequalities (Reid et al., 2019). A one health perspective is needed to control the increasing TB incidence at the global level, which is based on the interdependence of living being and environmental factors. This framework is essential for managing infections that can be transmitted between different species in the ecosystem. There are many classes of Mycobacterium, among which *Mycobacterium tuberculosis* causes TB in human beings, while other species of Mycobacterium such as *Mycobacterium bovis*, infect other animal hosts and aid zoonotic transmission (Olea-Popelka et al., 2017). The direct transmission of this pathogen to humans may occur through the use of dairy products and direct contact with cattle suffering from this disease (World Health Organization, 2017). The transmission is more commonly seen in low- or middle-income countries where disease control systems are limited, or animal health monitoring is very limited. The current One Health Framework adopted by the United Nations includes elimination as well as control strategies for zoonotic tuberculosis. The increase in the incidence of MDR and XDR has diminished the treatment of tuberculosis with the aid of traditional or alternative medicines. The resistance to the current line of antibiotics requires an immediate alternative intervention to treat and prevent the disease. Among the various alternative strategies, antimycobacterial peptides (AMPs) are emerging as an advanced treatment modality because of their lower ability to develop anti-mycobacterial resistance. Therefore, AMPs offer a green therapeutic option to conventional antimicrobial agents. In addition, peptide-oriented anti-mycobacterial therapies are biodegradable, due to which they have a lower impact on the environment. Hence, AMPs as anti-mycobacterial agents reinforce the focus of United Nation’s One Health perspective by supporting the SDG-3 goal of Good Health and Well Being. Apart from this, it will also help us to achieve the goal of a TB-free world by 2030 (Aslam et al., 2024).Till date BCG (*Bacille Calmette Guerin*) the only vaccine available for tuberculosis, is a live attenuated bacterial strain which is only effective in children and is ineffective in adults. The current therapy for the treatment of tuberculosis spans for around 6 to 9 months with four powerful anti-TB drugs, but upon its failure another 4 months of second line of anti-TB drugs constituting aminoglycosides, cycloserine, terizidione, ethionamide, protionamide, capreomycin and fluoroquinolones (Zhang et al., 2006; van den Boogaard et al., 2009). The second challenge is the existence of antibiotic persistent bacteria, which causes reactivation and active infection in some patients. The mycobacterial infections in these patients are harder to treat, and these persistent infections make up almost one third of the TB population. TB exists in two phases i.e. latent and active TB infection. As per WHO estimates, in 2024 approximately one-quarter of the global population (~2 billion people) living with latent TB infection, and among them, around 10 percent may develop active TB infection annually. In addition to the persistence of *M. tuberculosis* against anti-TB drugs, these drugs also interact with the antiretroviral therapy especially used for HIV treatment (McIlleron et al., 2007). The second line of drugs used against persistent tuberculosis is also associated with toxicity and low endurance (van den Boogaard et al., 2009). Hence the diminished efficacy of the BCG vaccine and the complications arising due to the anti-TB drug regime has led to the need for the development of novel anti-TB therapeutics with minimal drug interactions and the potential to shorten the duration of the current anti-TB therapy. Secondly, this therapeutics or compounds should be effective against the latent TB as well as the persistent *M. tuberculosis* population (Silva et al., 2016a). Natural compounds have been the prime source of medications since time immemorial and are often seen to have minimal side effects and drug interactions. In addition, these natural compounds have less chance of developing resistance and hence can prove beneficial in overcoming the current challenges faced by the TB drug regimen given to patients (Jha et al., 2012; Ageitos et al., 2017; Basu et al., 2022).

Contemporary literature has provided insightful summaries and information about the different classes of individually studied peptides; however, there remains a major need for convergence of structural chemistry of peptides with the actual clinical needs of treating TB. Thus, this review serves as a comprehensive overall design roadmap for the next generation of antimycobacterial peptides. By systematically correlating structure and activity we will address the many TB-related therapeutic gaps including eradication of PNP (latently infected) non-replicating PNP (NRP) bacilli, synergistic efficacy for mycobacterial pathogens that are MDR, and provide for maximized *in vivo* stability for individualized patients through targeted pulmonary route of administration. While previous reviews have systematically covered the general properties and mechanisms of antimicrobial peptides, the present work uniquely focuses on antimycobacterial peptides with specific activity against *M. tuberculosis* and related mycobacteria. This review offers a distinct comparison between human antimicrobial peptides like defensins, cathelicidins, and granulysin, and bacterial-derived non-ribosomal peptides such as cyclomarin A, rufomycin, and lacticin, as well as synthetic/engineered peptides including NZX and protegrins. In addition, this review provides a systematic way in which to prioritize peptide candidates in order to develop an adequate foundation for clinical testing of peptide drugs to directly treat tuberculosis.

## Drug resistance and immune invasion

**Figure 1:**
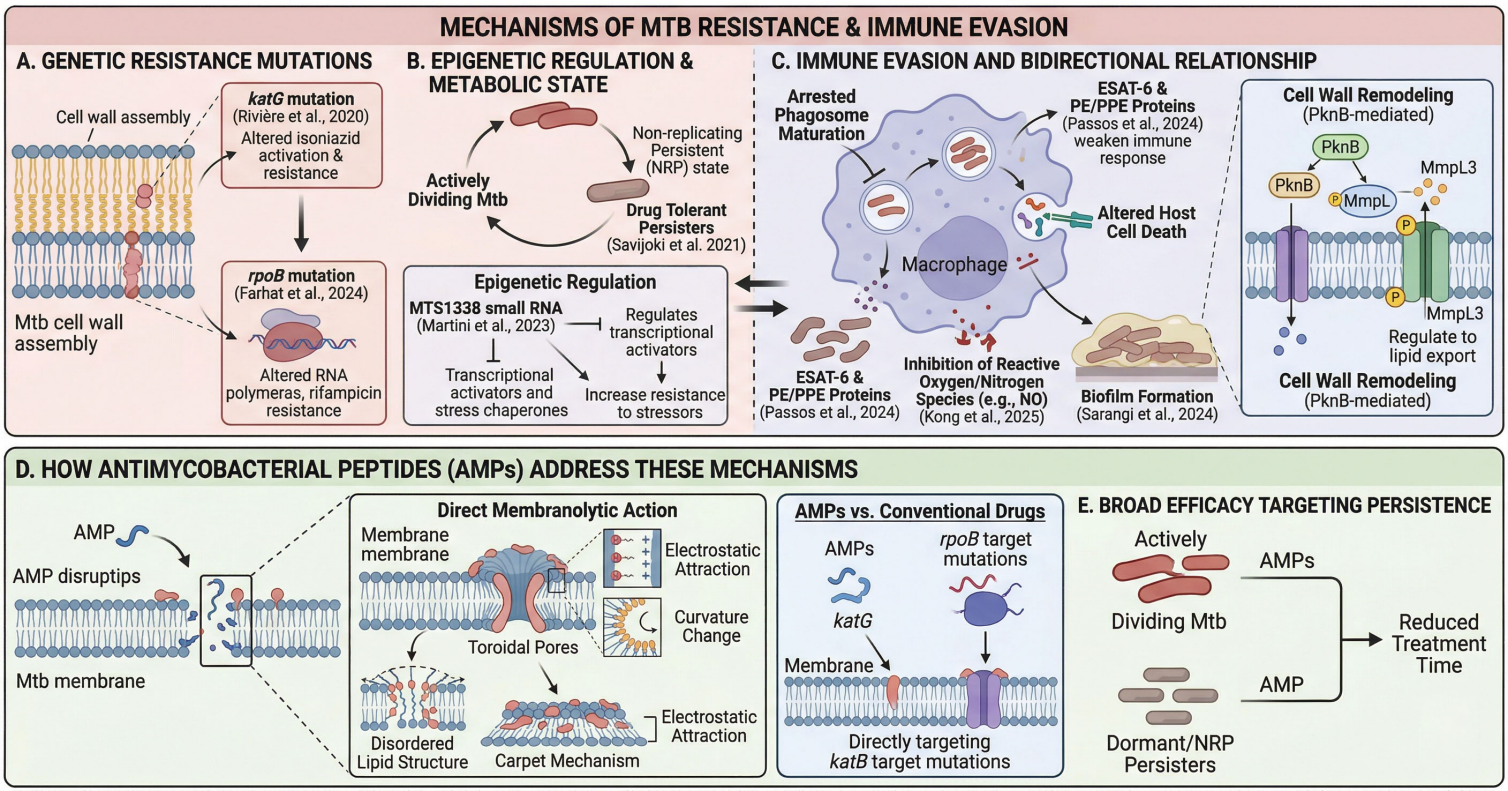
*Mycobacterium tuberculosis* (Mtb) resistance, immune evasion, and AMP therapeutic mechanisms. **(A)** Classical genetic resistance driven by target mutations **(B)** Epigenetic and metabolic shifts enabling stress-resistant, dormant (NRP) states **(C)** Immune evasion within macrophages **(D)** AMPs physically disrupting Mtb membranes to bypass classical target-based resistance **(E)** The broad efficacy of AMPs against both active and dormant Mtb populations to potentially shorten treatment.

The continued worldwide cases of tuberculosis (TB) can be attributed to the ability of *Mycobacterium tuberculosis* (Mtb) to use many types of methods to survive in the host and develop resistance toward antibiotic therapy. There are many things responsible for developing resistance to antibiotic therapy, such as cell wall remodeling, changing the way Mtb metabolically functions, and regulating Mtb in an epigenetic way. Each of these mechanisms is correlated with Mtb’s ability to evade the immune system (**Figures 1A–C**).

### The complex mechanisms of Mtb drug resistance and immune evasion

Genetic mutations that modify drug targets or activate drug pathways are primarily responsible for classical drug resistance in *Mycobacterium tuberculosis*. Mutations in the katG gene have been primarily attributed to isoniazid resistance for first-line drugs, while mutations in the rpoB gene have been attributed to rifampicin resistance (Rivière et al., 2020; Farhat et al., 2024). In addition to genetic modifications, *Mycobacterium tuberculosis*’ epigenetic regulation represents a complex toolkit for pathogen survival that allows it to adapt quickly to environmental stressors and develop antidrug resistance through rapid responses. For instance, MTS1338 small RNA has been shown to enhance isoniazid resistance by activating transcriptional regulators and stress-related chaperones in *M. tuberculosis* (Martini et al., 2023).

*Mycobacterium tuberculosis* (Mtb) actively changes its cell envelope in response to environmental pressures, such as drug treatment or starvation. For example, proteins, such as protein kinases (such as PknB), are involved in modifying the cell envelope of Mtb by regulating the phosphorylation of transport proteins (for example, MmpL3) to regulate the export of lipids. In addition, the metabolic reprogramming of Mtb allows it to survive in hostile environments and enter into a non-replicating persistent (NRP) state. This NRP state creates drug-tolerant persisters, which represent a significant barrier to treatment with antibiotics (Savijoki et al., 2021). The aforementioned adaptations have co-evolved and are intricately linked to immune evasion mechanisms, thereby creating a formidable barrier to effective treatment. The mechanisms of drug resistance and immune evasion have a bi-directional and cooperative relationship. *Mycobacterium tuberculosis* (Mtb) interferes with antigen presentation, arrests phagosome maturation, and alters host cell death signaling pathways (Pal et al., 2022). *Mycobacterium tuberculosis* (Mtb) modulates the host cells signal by releasing proteins like ESAT-6 and other proteins from the PE/PPE family, which weakens the immune response against the pathogen (Passos et al., 2024). Furthermore, Mtb inhibits the production of reactive oxygen and nitrogen species, including nitric oxide (NO), which are critical to the killing of the bacteria (Kong et al., 2025). Moreover, the biofilm of drug-resistant Mtb provide a refuge from the effects of antibiotics and immune cells (Sarangi et al., 2024).

Antimycobacterial peptides (AMPs) have a lower chance of developing antimicrobial resistance than antibiotics or other antimicrobial agents because AMPs can use multiple mechanisms to breach the cell membrane and kill bacteria, effectively evading most typical antimicrobial resistance mechanisms (**Figures 1D, E**). AMPs can disrupt cell membranes using the Toroidal Pore or Carpet Mechanisms through electrostatic attraction that induces changes in the curvature of the cell membrane, thereby creating disorder in the lipid structure. This rapid, physical disruption of the membrane by AMPs allows AMPs to circumvent the classic genetic mutations that often occur in some bacteria and alter the specific intracellular antimicrobial target(s). In addition, since AMPs are effective against both actively dividing and dormant populations of *M. tuberculosis*, they have the potential to significantly decrease the total treatment time and improve patient outcomes.

### Antimicrobial peptides as natural compounds

Antimicrobial peptides (AMPs) are natural compounds that generally span between 12–50 amino acids and are involved in the immune response for various organisms. Most of the antimicrobial peptides are positively charged and have a substantial portion of hydrophobic residues (Ageitos et al., 2017). These AMPs show a wide range of bioactivity against both Gram-positive and Gram-negative bacteria. The exhibition of this wide range of bioactivity may be due to the prevention of biofilm formation or by decreasing the quorum sensing factor in the bacteria (Silva et al., 2016a; Di Somma et al., 2020). In addition, AMPs like LL-37 act as immunomodulators and anti-inflammatory agents enhancing host immune system One of the major capabilities of AMPs is to exhibit a wide range of activity against a diverse array of microorganisms with low immunogenicity and specific selectivity to prokaryotic cell membranes (Waghu et al., 2013; Zhao et al., 2013). The advantage of using AMPs natural compounds over small molecules is their ability to show enhanced bioactivity and lower cytotoxicity. In addition, AMPs have a lower chance of developing antimicrobial resistance because they adopt multiple mechanisms to breach and kill bacteria, and hence can evade the resistance mechanisms of bacteria (Liu et al., 2021). In addition to membrane-based mechanism these peptides adopt non-membrane based mechanism such as interacting with and damaging intracellular targets such as DNA, RNA and protein to kill the microorganisms (Schmitt et al., 2011; Di Natale et al., 2020; Miryala et al., 2021; 2022). Hence, due to this ability to simultaneously target both the bacterial membrane and multiple essential intracellular processes through diverse structural scaffolds makes resistance development extremely difficult compared to single-target conventional antibiotics, positioning AMPs as highly promising therapeutic candidates especially against multidrug-resistant and extensively drug-resistant bacterial strains where traditional antibiotics fail.

### Structural factors linked with antimycobacterial activity in antimicrobial peptides

**Figure 2:**
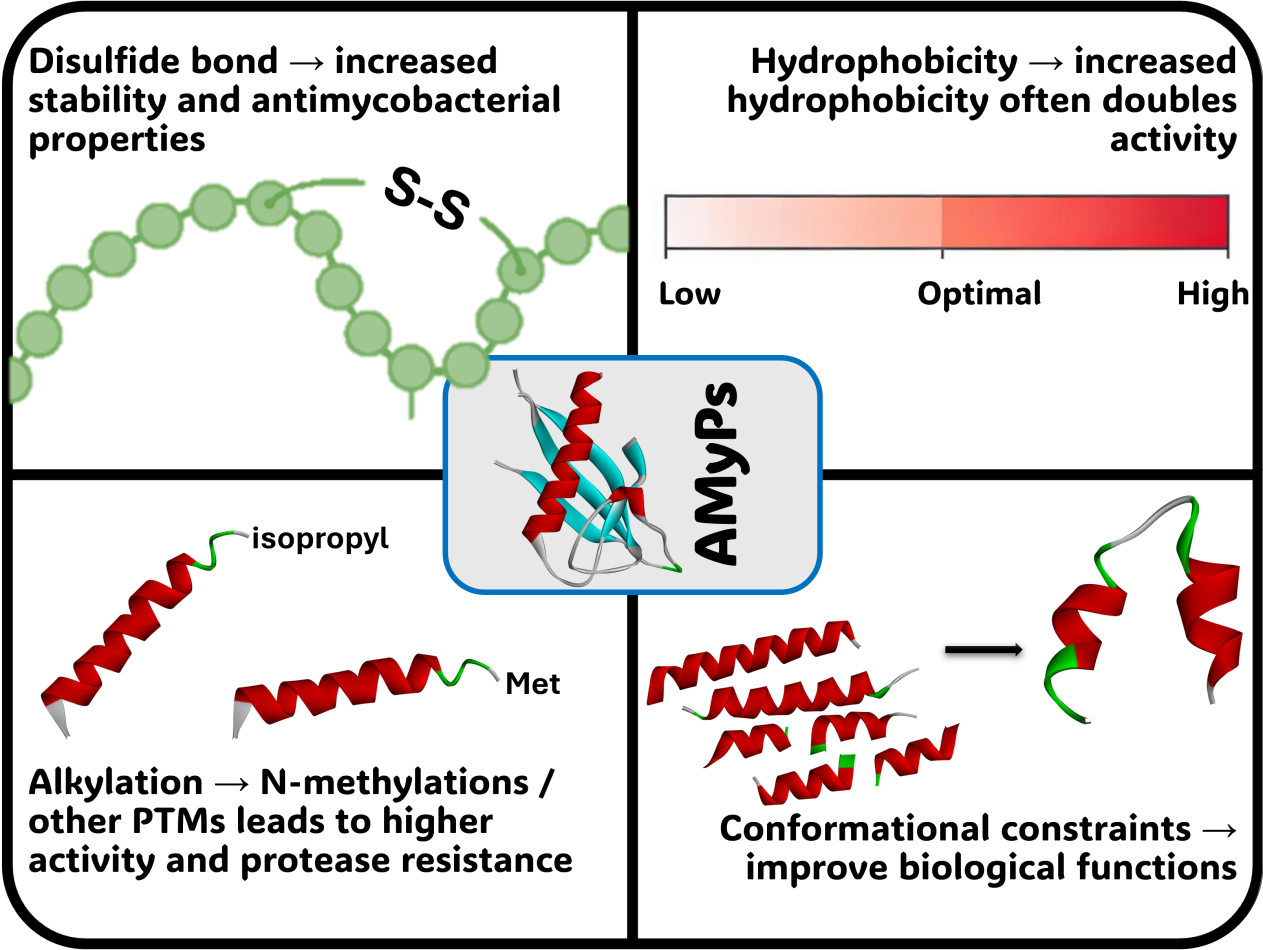
Influence of structural features and post-translational modifications on the antimycobacterial efficacy of AMPs. This image illustrates how specific structural elements—such as the inclusion of disulfide bonds for stability, an optimal balance between hydrophobic and polar regions, and cyclic rather than linear conformations—restrict flexibility and enhance the biological activity of antimicrobial peptides. Additionally, it highlights the role of post-translational modifications, such as N-methylation, in protecting peptides from proteolytic degradation and improving their overall antimicrobial and cytotoxic potency.

For a peptide to have antimycobacterial activity, a few factors need to be present. The antibacterial potency can be improved through implementing functional groups and structural changes, such as the inclusion of disulphide bonds, hydrophobicity, methylation etc (**Figure 2**) (Abedinzadeh et al., 2015). The disulphide bonds found in several antimycobacterial peptides, including VGF-1, HNP-1, lariatin A, NK-lysin, and protegrin (Andreu et al., 1999; Sharma et al., 2000; Xie et al., 2003; Iwatsuki et al., 2006; Ravishankar et al., 2016), usually contribute to peptide stability and, to a degree, improve their antimicrobial activity. For antimycobacterial function to be effective, it is essential that there be a proper balance between the hydrophobic and polar regions of the peptide (Chen et al., 2007). Studies have shown that peptides with greater hydrophobic properties can be better than peptide’s normal parenteral forms by almost two-fold (Abedinzadeh et al., 2015). In addition, the development of cyclic versus linear peptide design has been shown in previous research studies to limit the amount of flexibility, and this has led to improvements in the way peptides behave in a biological environment (Li et al., 2011). Comparative studies of kahalalide A and its related compounds show that when structures are restricted to a limited range of motion, their antimycobacterial activity is significantly enhanced relative to normal peptides (Suksamrarn et al., 2005). Lastly, post-translational modifications such as N-methylation improve the biological activities of peptides by protecting them from proteases. The inclusion of N-methylated phenylalanine within Pitipeptolides leads to improved antimycobacterial and cytotoxic properties (Montaser et al., 2011). This enhanced effectiveness is mirrored in compounds like ziziphine N and Q, which possess methoxy and N,N-dimethylated groups (Madanchi et al., 2020).

### Key factors affecting AMP activity

**Figure 3:**
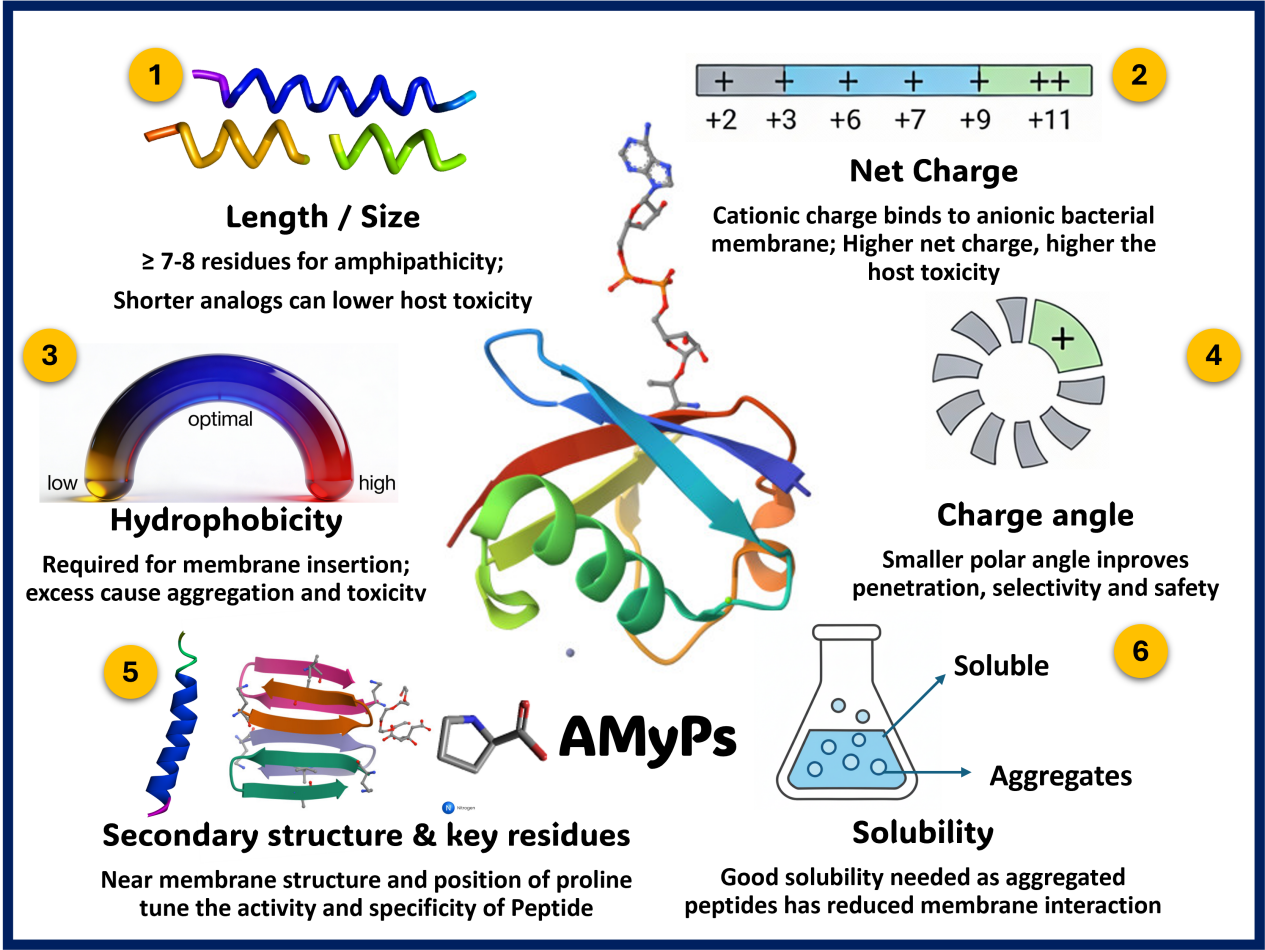
Key physicochemical determinants of antimicrobial peptide (AMP) activity. These schematic details the critical physical and chemical parameters required for effective AMP interaction with microbial membranes. Key factors depicted include peptide length, optimal hydrophobicity, amphipathicity (the presence of distinct water-compatible and incompatible regions), and a net positive charge (typically +2 to +11), which facilitates electrostatic attraction to anionic bacterial membranes. It also illustrates how secondary structural conformations (such as helices, sheets, or loops) and aqueous solubility dictate the peptide’s membrane-penetrating ability and target selectivity.

The primary function of AMPs is to interact with the membranes of microbes, and their overall efficacy is depended upon a number of characteristics such as size or the length of the peptide, net charge, charge angle, hydrophobicity, solubility, the secondary structure conformation, and amphipathicity (**Figure 3**) (Pushpanathan et al., 2013; Sd, 2014; Carratalá et al., 2020).

The AMPs to have an appreciable amphipathic structure, a minimum of seven or eight amino acids is required. Studies have revealed that shorter peptides of melittin and HP derivatives often exhibit decreased toxicity towards host cells but effectively maintain their antimicrobial properties, unlike their native form (Dathe et al., 2001; Park et al., 2007; Juba et al., 2015). In addition, the initiation of antimicrobial/antimycobacterial activity of the peptide also relies on the electrostatic interactions that occur between the peptide and the negatively charged moieties found on the bacterial membranes. Because of the presence of lysine and arginine residues, the majority of AMPs have a positive charge that falls between +2 to +11 (Pasupuleti et al., 2011; Sd, 2014). These positively charged peptides interact with the bacterial membranes because of their negative charge (Jiang et al., 2008; Mojsoska and Jenssen, 2015), but the presence of excessive positive charge has the potential to increase the toxicity in the host cells (AlMatar et al., 2018). Along with charge, hydrophobicity also plays a vital role in the interaction of these antimycobacterial peptides with the cell membrane. Each amino acid contributes to the peptide’s overall hydrophobicity, which affects membrane interaction (Rotem and Mor, 2009). It is important to note that while some level of hydrophobicity is required for antimicrobial activity (Khan et al., 2014), an excess of it can lead to the aggregation of peptides, which in turn reduces their ability to exhibit antimycobacterial function (Yin et al., 2012). Therefore, the presence of excessive hydrophobic moieties, due to their affinity for aggregation, could be potentially detrimental to mammalian cells (Wieprecht et al., 1997; Chen et al., 2007).

Amphipathicity, characterized by the presence of both water-compatible and water-incompatible areas, is a key aspect of membrane binding AMPs, and it is considered even more critical than just hydrophobicity (Chen et al., 2005b). In helical AMPs, the angle formed by positively charged residues affects their amphipathicity. Studies have shown that smaller charge angles affect membrane penetration ability and also the anti-bacterial selectivity for the AMPs (Uematsu and Matsuzaki, 2000; Vermeer et al., 2012).The secondary structure (helix, sheet, or loop) that AMPs adopt near the membranes influences their effectiveness and selectivity (Dathe and Wieprecht, 1999; Xie et al., 2011). Proline residues are key players in membrane crossing and target specificity (Park et al., 2000; Cutrona et al., 2015), and the position in which they appear in the sequence is a crucial factor (Fernández-Vidal et al., 2007). In addition, AMPs must remain dissolved in aqueous environments to reach their targets (AlMatar et al., 2018). As peptides aggregate, they become less able to interact with membranes, and their solubility diminishes, which is an essential consideration in the process of designing and optimizing synthetic AMPs (Matsuzaki et al., 1995).

## Antimycobacterial peptides and its classifications

Most of the antimycobacterial peptides are cationic and amphipathic in nature. These peptides have the ability to breach the mycobacterial cell wall and interact with various mycobacterial intracellular organelles and enzymes. AMPs can be classified based on their source of origin, activity, structure, and amino acids. The subsequent sections enlist the various AMyPs that have shown antimycobacterial activity.

## Human innate immune peptides

Our body releases an array of antimycobacterial peptides upon mycobacterial infection. These AMyPs include Cathelicidins, Defensins (α and β), Iron-Regulatory and Granule Proteins like Lactoferrin, Hepcidin, Granulysin and Azurocidin and Enzymatic Host Defense Peptides like Elastases.

### Cathelicidins/LL-37

The LL-37 is a cationic peptide derived from the C-terminal region of the human cathelicidin precursor (hCAP-18)—is a 37-amino-acid amphipathic peptide (**Table 1**), which is preponderantly stored in neutrophil granules and other immune cells, then released and proteolytically processed into its active form (Kuroda et al., 2015). This peptide shows potent anti-mycobacterial activity, particularly against *Mycobacterium tuberculosis* (strain H37Rv) and *Mycobacterium bovis* inside infected macrophages. The treatment with LL-37 reduced viable bacterial load by 60% (Rivas-Santiago et al., 2013; Teng et al., 2015; Oliveira et al., 2021). The reported minimum inhibitory concentration (MIC) for *M. tuberculosis* H37Rv for LL-37 has been reported to be approximately 5 µg/mL (Teng et al., 2015; Oliveira et al., 2021). Mechanistically, LL-37 is thought to kill mycobacteria primarily via disruption of the mycobacterial membrane barrier by membrane destabilization, pore formation leading to loss of membrane function and bacterial death (Reinholz et al., 2012; Teng et al., 2015) In addition to direct bactericidal activity, LL-37 also exhibits immunomodulatory properties. In infected macrophages, LL-37 treatment modulates cytokine responses, increasing early pro-inflammatory mediators (e.g., IL-1β, TNF-α) and later anti-inflammatory cytokines (e.g., IL-10, TGF-β) suggesting that LL-37 helps orchestrate a balanced immune response during mycobacterial infection (Teng et al., 2015; Torres-Juarez et al., 2015; Oliveira et al., 2021). Collectively, the data support that LL-37, via combined membranolytic and immunomodulatory mechanisms, represents a promising host-derived effector against *M. tuberculosis* and related mycobacteria.

**Table 1:** Comparative structural and functional properties of α-Defensins.

Peptide	Length	Difference in sequence	MIC (µg/mL)	Rate of activity
HNP-1	30aa	Reference sequence ACYCRIPACIAGEAAYGTCIYQGALWAFCC	2.5	High activity
HNP-2	29aa	Lacks N-terminal Ala	5.0	Similar to HNP-1
HNP-3	30aa	N-terminal Ala→Asp substitution	8.0	Lowest activity

### Defensins

These AMyPs for TB are a set of cationic and cysteine-rich peptides classified according to their structure into alpha, beta. These are diverse in their amino acid sequences and show a great amount of variation in their mechanisms of action at membrane and intracellular levels (Faurschou et al., 2005).

### α-defensins or human neutrophil peptides

α-defensins are cationic molecules predominantly stored in the azurophilic granules of polymorphonuclear neutrophils, where they serve as an early effector of innate immunity (Sharma et al., 1999). The peptides have 29–30 amino acids (**Table 1**) and their structure is stabilised by three conserved intramolecular disulfide bonds (Cys1-Cys6, Cys2-Cys4, Cys3-Cys5). The crystallographic structure of HNPs reveals that these peptides have, a β-sheet-rich architecture typical of α-defensins (Sharma et al., 1999). Human neutrophil peptide-1 (HNP-1) is a particular type of cationic α-defensin that is released by the azurophilic granules of human polymorphonuclear neutrophils (PMNs) (Miyakawa et al., 1996). HNP-1 plays an important role in initially limiting intracellular infectious agents. HNP-1 forms a compact pattern, which is stabilized by disulfide bonds and has a resolved structure (PDB: *2PM5*) similar to other human neutrophil defensins (Kalita et al., 2004). HNP-1 has the ability to inhibit the growth of *Mycobacterium tuberculosis* in many *in vitro* and *in vivo* models. As shown by minimum inhibitory concentration (MIC) studies, HNP-1 inhibits the growth of bacteria in large quantities. Colony-forming unit (CFU) studies demonstrate that HNP-1 significantly reduces the number of viable *M. tuberculosis* (Miyakawa et al., 1996). HNP-1 is shown to decrease the intracellular load of *Mycobacterium tuberculosis* by more than 90% in macrophages infected with this organism in a laboratory setting. In addition, to its effect at the level of the host cell surface, α-defensins such as HNP-1 may also participate in modulating signalling pathways associated with innate immunity through stimulation of phagosomal maturation or production of antimicrobial substances that act in concert with direct microbicidal activity. The studies also point to the role that cationicity and β-sheet abundance play in the interaction of the peptide with the mycobacterial plasma membrane, providing a basis for destabilising it and depleting the bacilli of energy sources (Fu, 2003). The antimicrobial activity of HNP-1 is diverse due to its ability to kill bacteria and its unique capacity to alter the body’s response to an infection by helping modulate various factors related to the immune system. Because of these properties, HNP-1 represents both a natural defense mechanism against disease and a potential template for developing new peptides to treat tuberculosis. Similarly, Human Neutrophil Peptide-2 (HNP-2) is a cationic α-defensin located in the azurophilic granules of Polymorphonuclear Neutrophils. These peptides are released during the early phases of antibacterial defence. The crystallographic studies have clearly shown that the β-sheets with disulfide bridges show characteristics similar to neutrophil defensins. This fold provides resistance to proteolytic degradation and also enables high-affinity binding to microbial membranes (Lourenzoni et al., 2007). The ability to reduce extracellular bacillary proliferation is determined by minimum inhibitory concentration (MIC) assays and colony-forming unit (CFU) enumeration (Fu, 2003). *In vitro* studies have shown that HNP-2 substantially reduces the mycobacterial burden in macrophages, thereby demonstrating its proficiency in unfavourable conditions (Fu, 2003). HNP-2 works with macrophage immunological pathways to enhance the bactericidal effect. This can occur by increasing oxidative stress, by regulating cytokine release, or by increasing the phagosomal maturation rate. Several structural analyses have shown that small sequence divergence in HNP isoforms has created a strong action of HNP-2 against anionic cell envelopes that fine tune the electrostatic distribution among them. Finally, according to the crystallographic studies, HNP-3 is a type of α-defensin which shows a cysteine-braced β-sheet framework. The dense disulfide fold not only provides it with stability but also attracts the negatively charged membranes of pathogens (Lourenzoni et al., 2007). Studies carried out on HNP-3 for bacillary proliferation show similar like HNP-2. It was seen in MIC assays that viable organisms were diminished in CFU-based enumeration, and intracellular survival of Mtb within infected macrophages was markedly reduced (Fu, 2003). Mechanistic studies have suggested that HNP-3 may disrupt mycobacterial membranes by electrostatic forces. For promoting efficient interaction, innate immune signalling cascades are also escalated at the same time (Chen et al., 2001). Sequence divergence studies have shown that an arginine-enriched motif distinctive to HNP-3 can enhance selective binding to lipid-rich mycobacterial envelopes, enhancing its bactericidal potency (Corbett and Berger, 2005).

### β-defensins or human β-defensins

β-Defensins, also known as Human β-defensins, are another human defensin which are generally expressed in both leukocytes and epithelial cells lining the respiratory tract, positioning them as the first line of defense against inhaled *M. tuberculosis* bacilli. These constitute of 36–49 amino acid sequences with conserved three disulfide bonds (Cys1-Cys5, Cys2-Cys4, Cys3-Cys6) different from the α-Defensins (Gonzalez-Curiel et al., 2011). Human β-defensin-1 (HBD-1) is a positively charged and cysteine rich antimicrobial peptide, produced continuously by epithelial linings and leukocytes. It acts as a primary barrier to invading pathogens. While its antimicrobial activity has been known for decades, recent research has pivoted toward its role as a diagnostic and prognostic biomarker for diseases like tuberculosis, acute-on-chronic liver failure, and certain urogenital cancers (Álvarez et al., 2018; Mani et al., 2019). As per the crystallographic studies, it has a β-sheet architecture linked by three disulfide folds. The stability of the microbial membrane and its tolerance to high salt concentration is provided by disulfide folds (Fattorini et al., 2004). Antimycobacterial effects of HBD-1 are owed to the successful growth inhibition in MIC assays and inhibition of bacillary proliferation has been confirmed through CFU-based quantification (Lourenzoni et al., 2007). Recent investigations found that HBD-1 functions through mechanisms extending beyond mere membrane perturbation. Under oxidative or inflammatory stress, epithelial cells upregulate defensin-linked pathways, enhancing the microbicidal activity of HBD-1. A distinctive arginine-rich cluster at the C-terminus of HBD-1 increases electrostatic attraction toward the anionic mycobacterial envelope, which may account for its microbicidal activity (Chen et al., 2005a). These properties define HBD-1 as a promising molecular scaffold for the rational design of next-generation host-directed interventions against tuberculosis. Human β-defensin-3 (HBD-3) is also a positively charged and cysteine-rich antimicrobial peptide which has an arginine rich C terminal. This arginine rich C terminal provides strong binding to the pathogen. Its crystallographic structure shows triple disulfide β-sheet fold of β-defensins. HBD-3 is set aside from others due to its enhanced surface charged density (Pazgier et al., 2007). It can successfully inhibit mycobacterial growth in MIC assays, thus confirming its antimycobacterial effects. Moreover, its inhibition of bacillary proliferation has been confirmed through CFU-based quantification (Corrales-Garcia et al., 2013). It also has the ability to enhance the host’s capacity to control intracellular mycobacteria by acting as immune modulating peptide. Studies have shown that HBD-3 can boost chemotactic responses, promote cytokine and chemokine release, and even stimulate dendritic cell activation, which could synergise with antimicrobial pathways and indirectly restrict Mtb survival. When taken together, HBD-3 acts as one of the strongest and adaptable β-defensins which can combine antimycobacterial actions with immunomodulatory effects to counter the effects of Mycobacterium. Research focuses on protease-resistant analogs and biomaterial-based delivery systems (Nguyen et al., 2023). Human β-defensin-4 (HBD-4) made by the leukocytes and epithelial linings, is cationic and cysteine rich in nature whose production increases during inflammation. Studies also explore chimeric defensins and biomarker applications (Zhang et al., 2025). The structural analysis has resolved that the mature peptide – EFELDRICGYGTARCRKKCRSQEYRIGRCPNTYACCLRKWDES - folds into a three-disulfide, β-sheet scaffold of β-defensins, which generates a densely charged molecular surface that facilitates strong electrostatic interactions with microbial membranes. *In vitro* assays confirm that HBD-4 can decrease Mtb proliferation, thereby confirming its growth inhibition (Lourenzoni et al., 2007). The role of HBD-4 appears to extend beyond microbial membrane disruption. HBD-4 has been linked to the regulation of innate immune responses, including the development of early cytokine dynamics and the enhancement of chemotactic signals. Due to its robustness and proven antimycobacterial efficacy, it has been considered a viable option for host directed treatment for tuberculosis.

### Granulysin

Granulysin is an antimicrobial peptide with a high molecular weight, cationic charge, and is primarily produced by cytotoxic T lymphocytes. Natural killer (NK) cells produce a smaller amount of granulysin. Granulysin is a critical component of the adaptive immune system because it plays a role in eliminating both infected host cells and invading microbes. When a T-cell is activated, granulysin is released from the cytotoxic granule, and it works together with perforin and granzymes to destroy cells infected with intracellular pathogens and to attack the invading microbes directly. The granulysin peptide is initially produced in a larger precursor form of 15 kDa and is subsequently cleaved to produce the biologically active 9 kDa form (**Table 1**). Granulysin has a compact, disulfide-stabilized, saposin-like structure (PDB: *1L9L*) that allows it to bind and insert into lipid-rich membranes (Mueller et al., 2011). Granulysin is the first T-cell-derived antimicrobial protein identified and has a wide range of anti-mycobacterial actions that make it possible for it to kill both intra- and extra-cellularly present *Mycobacterium tuberculosis*. The primary mechanism of action for granulysin is complex and may occur through a variety of mechanisms including creating pores in the membrane of mycobacterium; causing osmotic ruptures in the cell membrane; and disrupting the normal metabolic pathways necessary to the growth of mycobacterium which ultimately leads to the death of the bacteria. Importantly, *in vivo* studies support the physiological activity of granulysin, where increased granulysin levels are associated with a significantly decreased pulmonary bacillus burden in mouse model of tuberculosis. In summary, granulysin plays an important role in T-cell-mediated antimicrobial defence. In addition, it has been shown to behave as both a cytolytic molecule and as a direct antimicrobial agent against resistant organisms such as *M. tuberculosis* (Toro et al., 2006). Research focuses on granulysin-derived peptides and immunotoxin-based therapies for multidrug-resistant cancers and chronic acne (Guerrero-Ochoa et al., 2022). Overall, the high cytolytic activity and significant *in vivo* effectiveness of granulysin position it as an important multifunctional immune effector and support its relevance to protecting the host from mycobacterial infection.

### Lactoferrin

Lactoferrin is a glycoprotein with multiple functions and is found throughout all mammalian body fluids, such as saliva and breast milk, as well as in tears and respiratory fluids. Lactoferrin’s primary role is in mucosal immunity and early defense against microbial attacks. Lactoferrin has many known properties, such as the ability to bind metals and modulate the immune response. However, it also contains a region that appears to be a peptide useful in the development of antimicrobial peptides (**Table 2**). From a structural perspective, the peptide has a very compact shape, as seen from the crystallographic data (PDB: *1Z6V*), allowing it to interact with the membranes of microorganisms and receptors of the immune system (Nicolas et al., 2002). The evidence continues to build showing that lactoferrin has a notable level of anti-mycobacterial activity, showing lactoferrin’s ability to limit or minimize the spreading of *Mycobacterium tuberculosis* to the liver, lower CFU loads in lungs and stop the systemic distribution of *Mycobacterium tuberculosis* to infected hosts (Sow et al., 2007). The ability of lactoferrin to enhance the effectiveness of the BCG vaccine may be attributed to its ability to regulate antigen-presenting cell (APC) functions, stimulate APA-driven responses, and augment Th1-mediated immune responses. These mechanisms may act together to improve immune function to the level necessary to protect against *M. tuberculosis* infection (Sow et al., 2011; Layoun and Santos, 2012). These observations collectively point out that lactoferrin is a highly versatile innate immune molecule that has characteristics and functions of immunomodulation as a way of contributing to its role in protection against mycobacterial infections.

**Table 2:** Comprehensive overview of antimycobacterial peptides, their sources, structural identifiers, and mechanisms of action.

Sl. no.	Peptides	Sequence	Source	Anti-mycobacterial properties	PDB ID
1	Cathelicidins/LL-37 [CATIONIC]	LLGDFFRKSKEKIGKEFKRIVQRIKDFLRNLVPRTES	Pre-dominant in neutrophiles	Membrane barrier, disruption or pore formation	2K6O
2	Granulysin [CATIONIC]	GRDYRTCLTIVQKLKKMVDKPTQRSVSNAATRVCRTGRSRWRDVCRNFMRRYQSRVIQGLVAGETAQQICEDLR	CTL cells	Decreasing the bacilli load in the lungs (*in vivo* mice model); cytolytic against intracellular and extracellular Mtb	1L9L
3	Lactoferrin [CATIONIC]	GRRRRSVQWCAVSQPEATKCFQWQRNMRKVRGPPVSCIKRDSPIQCIQA	Present in mammalian secretions	Decreases dissemination of bacteria to liver; lowerCFU count in lung	1Z6V
4	Hepcidin [CATIONIC]	DTHFPICIFCCGCCHRSKCGMCCKT	Macrophages and liver hepatocytes	Restricts mycobacterial growth by inhibiting the release of recycled iron by macrophages; structural damage to Mtb	1M4F
5	Lacticin 3147 [CATIONIC]	AADhbNDhbFAL ADYWGNNGAWAA buLAbuHEAMAWAK	*Lactococcus lactis*	Peptidoglycan synthesis inhibition; possess strong inhibitory action than rifampicin MIC90 (Mtb-H37Rv): 7.5 μg/ml MIC90 (M. avium-*in vitro*): 60 μg/ml MIC90 (M. kansasii-*in vitro*): 15 μg/ml	–
6	NZX [CATIONIC]	GFGCNGPWNEDDLRCHNHCKSIKGYKGGYCAKGGFVCKCY	Plectasin (derived from the fungus Pseudoplectania nigrella)	Concentration-dependent inhibition of H37Rv and MDR isolates; reduction in number of CFUs present in the lungs similar to RIF.	6K50
7	Protegrins [CATIONIC]	RGGRLCYCRRRFCVCVGR	Porcine leukocytes	68.4% CFU reduction against Mtb H37Rv at 64 μg/ml and against MDR strains at 128 μg/ml	1PG1
8	HNP-1 [CATIONIC]	ACYCRIPACIAGERRYGTCIYQGRLWAFCC	Azurophilic granules of polymorphonuclear neutrophils	Plasma membrane binding followed by permeabilization	3GNY
9	HNP-2 [CATIONIC]	CYCRIPACIAGERRYATCIYQGRLWAFCC	Azurophilic granules of human polymorphonuclear neutrophils	Inhibit Mtb growth in MIC determination, CFU assays, and reduction of the mycobacterial load in macrophages infected *in vitro*.	1ZMH
10	HNP-3 [CATIONIC]	DCYCRIPACIAGERRYGTCIYQGRLWAFCC	Azurophilic granules of human polymorphonuclear neutrophils	inhibit Mtb growth in MIC determination, CFU assays, and reduction of the mycobacterial load in macrophages infected *in vitro*.	1DFN
11	HBD-1 [CATIONIC]	DHYNCVSSGGQCLYSACPIFTRIQGTCYRGRARCCR	Human leukocytes and epithelial cells	Inhibit Mtb-growth in MIC determination	2PLZ
12	HBD-3 [CATIONIC]	GIINTLQKYYCRVRGGRCAVLSCLPKEEQIGKCSTRGRKCCRRKK	Human leukocytes and epithelial cells	Inhibit Mtb-growth in MIC determination	1KJ6
13	HBD-4 [CATIONIC]	EFELDRICGYGTARCRKKCRSQEYRIGRCPNTYACCLRKWDES	Human leukocytes and epithelial cells	Inhibit Mtb-growth in MIC determination	5KI9
14	Ub2 [CATIONIC]	GAMGSMQIFVKTLTGKTITLEVEPSDTIENVKAKIQDKEGIPPDQQRLIFAGKQLEDGRTLSDYNQKESTLHLVLRLRGGMQIFVKTLTGKTITLEVEPSDTIENVKAKIQDKEGIPPDQRLIFAGKQLEDGRTLSDYNIQKESTLHLVLRLRGG	Human lysosome	Disrupts microbial cell membrane	4ZQS
15	Dermcidin [ANIONIC]	SSLLEKGLDGAKKAVGGLGKLGKDAVEDLESVGKGAVHDVKDVLDSVL	Sweat glands of human	Inhibits cell wall biosynthesis and binds to bacterial envelope	2KSG
16	NP-1 [CATIONIC]	VVCACRRALCLPRER RAGFCRIRGRIHPL CCRR	Rabbit (Oryctolagus cuniculus) leucoc (noytes	Disrupts the microbial cell membrane	
17	Indolicidin [CATIONIC]	ILPWKWPWWPWRR	Bovine neutrophils	Inhibit Mtb-growth in MIC determination	1G89
18	Capreomycin [CATIONIC]	Cyclo (Dpr‐bUdAla‐ Cap‐Dpr‐Ala) v	Streptomyces capreolus	Inhibits protein synthesis	
19	Rufomycin I/llamycin A [neutral cyclic non-ribosomal peptide]	Chain B_Rufomycin I|Streptomyces atratus (1893) XLYAXLX	*Streptomyces* sp. (MJM3502) *Streptomyces atratus* (NRRL B-16927)	Inhibits the action of ClpC1	6CN8
20	Cyclomarin A [neutral, hydrophobic cyclic non-ribosomal peptide.]	WLAFVLV	*Streptomyces* sp. (CNB-982)	Inhibits the action of ClpC1	3WDC
21	GranF2 [CATIONIC]	VCRTGRSRWRDVCRNFMRRYQSR	Synthetic, derived from granulysin bactericidal protein	Disrupts microbial cell membrane and induces apoptosis of mammalian cell	

### Dermcidin

Dermcidin is an antimicrobial peptide that plays a role in the first line of defense against Mycobacterial infections. It is 48 amino-acids long, very stable at high concentrations of salt and at a wide range of pH-values. Analysis of its structure shows that Dermcidin possesses a unique 3D shape, enabling it to attach to many different kinds of microorganisms. In addition to binding to the exterior of the Mycobacteria, Dermcidin interferes with their normal functioning by slowing down their production of cell walls (Schittek et al., 2001; Banerjee and Gohil, 2016). The absence of reported cytotoxicity associated with dermcidin is related to its compatibility with host tissues and to the fact that dermcidin has a physiological role in skin defensive mechanisms. Dermcidin and processed fragments derived from dermcidin have also been found to have broad-spectrum antimicrobial effects, and to show sustained persistence on the skin’s surface, providing evidence for the evolutionary adaptation of dermcidin and its processed fragments as endogenous antimicrobial agents (Schittek and Hipfel, 2001; Zasloff, 2002; Brogden, 2005).

### Hepcidin

Hepcidin, an essential link between the innate immune system and iron regulation, is a small peptide produced mainly by the liver but also by macrophages during infection and immune response (Meher et al., 2006). Structural studies have revealed that hepcidin forms a compact, stable structure composed of multiple disulfide bonds (PDB: 1M4F), allowing it to retain its structure in body fluids and interact with metal ions or microbes 50 or more. In the context of mycobacterial infection, hepcidin exhibits anti-mycobacterial activity by limiting the release of recycled iron from macrophages, thereby depriving intracellular pathogens such as *Mycobacterium tuberculosis* of a critical micronutrient essential for their growth (Purdy and Russell, 2007). Experimental evidence shows that upon infection of macrophages, hepcidin expression is induced and localizes to phagosomes containing mycobacteria, where it can have a direct antimicrobial effect (Purdy and Russell, 2007). In context to this, in a recent study using a macrophage activation model, endogenously induced hepcidin co-localised within phagosome-resident *M. tuberculosis* inhibited the intracellular growth, reinforcing the physiological relevance of hepcidin as a host defence effector within infected macrophages (Sow et al., 2007; Park et al., 2025). Moreover, beyond mycobacteria, hepcidin’s broad antimicrobial functionality has been explained across various bacterial species for both Gram-positive and Gram-negative — in non-mammalian vertebrates, illustrating the evolutionary conservation of its cationic antimicrobial mechanism, and suggesting that its disulfide-stabilised, cationic structure is a general-purpose weapon in innate immunity (Lin et al., 2014; Ahmadi Badi et al., 2024).

### Non-ribosomal and bacterial peptides

#### Cyclomarin A

Cyclomarin A is a potent anti-tuberculosis and anti-malarial cyclic non-ribosomal peptide produced by the bacterium *Streptomyces* sp. CNB-982, that has lipophilic properties and a rigid macrocyclic structure that makes it highly hydrophobic. The preclinical stage data shows that it specifically interferes with the proteostasis system of mycobacterial cells by deregulating the ClpC1-ClpP protease complex (Kazmaier and Junk, 2021; Taylor et al., 2022). The structure of cyclomarin A has been studied using various techniques, including X-ray crystallography. Researchers have determined that cyclomarin A contains a small peptide sequence, WLAFVLV (Chain B), and its major target in *Mycobacterium tuberculosis* is ClpC1, an ATP-dependent subunit of the Clp protease complex. Cyclomarin A is found to bind at the N-terminal regulatory domain of ClpC1, inhibiting the ATP-dependent chaperone function of ClpC1 which is crucial for maintaining bacterial protein quality when under stress. The anti-mycobacterial properties of Cyclomarin A, along with other members of its family, come from destabilising the ClpC1 - ClpP1P2 proteolytic complex, leading to the breakdown of essential substrates involved in this process (Schmitt et al., 2011; Noble et al., 2013). Subsequent synthetic and biosynthetic efforts broadened the chemical diversity of cyclomarin analogues, reinforcing their designation as privileged scaffolds for selective ClpC1 inhibition (Barbie and Kazmaier, 2016; Kiefer et al., 2019). Broader evaluations of ClpC1 inhibitors have underscored their therapeutic promise as next-generation interventions against multidrug-resistant tuberculosis (Koul et al., 2011; Kalo et al., 2012). Cyclomarin A is a prime example of how the complexity of its structure as a natural product leads to its exceptionally strong selectivity for an antimycobacterial mode of action, making Cyclomarin A the best lead for future tuberculosis drug development.

#### Rufomycin I/llamycin A

Rufomycin I (also referred to as Illamycin A) is a neutral cyclic non-ribosomal peptide produced by the genus Streptomyces, including *Streptomyces atratus* (NRRL B-16927) and the species Streptomyces sp. MJM3502. This has been shown to produce secondary metabolites that have structural complexity and show activity against mycobacteria. Rufomycin occupies a regulatory site of ClpC1, thereby inhibiting the ATP hydrolysis driven proteolytic cycle, which is an important mechanism for the survival of *Mycobacterium tuberculosis* under stress. Research on rufomycin analogues has shown that these compounds disrupt ClpC1 function and demonstrate a high degree of specificity for drug resistant Mtb strains, inhibiting their growth (Khalil et al., 2014; Wolf et al., 2019). A broad analysis of many (non-ribosomal) peptides has shown rufomycin analogs to be stable in an aqueous solution and also carry properties that can make them easy to cross into cells and allow them to interact with a variety of complex cellular proteins (Süssmuth and Mainz, 2017). In addition, other studies on rufomycin analogs for drug discovery have illustrated their utility as potential members of the ClpC1 inhibitors, a new class of anti-TB agents designed to address drug-resistant TB and extensively drug-resistant TB (Koul et al., 2011). Collectively, the available data suggest that rufomycin is a unique chemical entity for which new molecules will continue to be generated based on either structural or mechanistic modifications to existing compounds.

#### Lacticin 3147

Lacticin 3147, a potent bacteriocin from *Lactococcus lactis*,is a cationic peptide that has emerged as a promising antimicrobial peptide with notable activity against pathogenic mycobacteria. Its dual-peptide architecture and lipid II–targeting mechanism have been well characterised biochemically (Cotter et al., 2013). By disrupting peptidoglycan biosynthesis through lipid II binding and pore formation, Lacticin 3147 acts at a site distinct from conventional tuberculosis drugs, and intriguingly, its antimycobacterial potency can exceed that of rifampicin (Asaduzzaman and Sonomoto, 2009). Some work has shown synergistic activity with other antimicrobial peptides and stability under physiological conditions (Hall et al., 2015).

#### Capreomycin

Capreomycin is derived from the bacterium *Nonomuraea* sp. MJM5123 and has a unique multi-chain structure that distinguishes it from other antibiotics. It is an extremely potent inhibitor of *Mycobacterium tuberculosis* that is resistant to other commonly used anti-tuberculosis agents. The peptide of the capreomycin molecule is made up of a short four amino acid motif (LVAWG) in the peptide chain C, and the biological activity of capreomycin is closely associated with its interaction with the Clp protease ATP-binding subunit ClpC1 of *M. tuberculosis*. Once capreomycin binds to ClpC1, it inhibits the proteolytic activity of ClpC1, leading to defects in essential metabolic and environmental stress-response pathways. The evidence supporting the biochemical and structural basis of this process is strong (Gao et al., 2014, 2015).

#### Protegrins

Protegrins are cationic and cysteine rich peptides that are mainly found in porcine leukocytes. These peptides play an important role in innate immune defence against a broad spectrum of pathogens (Goldman et al., 1997). The secondary structure of the peptide is composed of β-hairpin structure which is stabilised by disulfide bonds. This stable structure lends the peptide to efficiently interact with the cell membranes of bacteria and breach the cell surface, thus exhibiting potent antimycobacterial activity (Goldman et al., 1997; Moulahoum et al., 2020). Functionally, protegrins exhibit potent anti-mycobacterial activity. *In vitro* studies demonstrate a 68.4% reduction in colony-forming units (CFUs) of *Mycobacterium tuberculosis* H37Rv at 64 μg/mL, while multidrug-resistant (MDR) *M. tuberculosis* strains require slightly higher concentrations (128 μg/mL) to achieve similar bacterial inhibition (Chen et al., 2009). Mechanistically, protegrins primarily act through membrane disruption, creating pores in the bacterial cell envelope that compromise integrity and lead to rapid bacterial death. Their amphipathic β-hairpin structure is particularly suited for inserting into lipid bilayers and disrupting membrane potential. Current research focuses on designing safer protegrin analogs as antimicrobial scaffolds (Rizzetto et al., 2025).

#### Indolicidin

Indolicidin, a short cationic host-defense peptide derived from bovine neutrophils, has a unique tryptophan-rich structure known for its ability to disrupt cell membranes. It has been studied using crystallographic techniques, which show that Indolicidin has a flexible, amphipathic structure that allows it to efficiently integrate into the lipid bilayers of microorganisms. It is also very effective against mycobacteria and has been shown to inhibit the proliferation of *M. tuberculosis* in MIC assays by disrupting mycobacterial membranes and potentially targeting intracellularly (Portell-Buj et al., 2019). The work done as part of the current studies on Indolicidin has demonstrated that this peptide contains an unusually high concentration of Tryptophan and it is also capable of exhibiting a broad range of antimicrobial activity when compared to other members of the cathelicidin family (Selsted et al., 1992). Along with other articles, the Indolicidin (and other Cathelicidins) dual function in killing microbes and modifying the immune system has been further emphasised in most publications. It is now well understood that Indolicidin is also capable of influencing cell signalling via cytokine receptors that play a role in influencing the immune system and enhancing phagocytosis (Andreu and Rivas, 1998). Recent articles have also provided evidence to support the potential application of Indolicidin in treating drug-resistant pathogen infections and highlighted its ability to disrupt biofilm formation as well as retain activity against a wide variety of bacteria with complex mechanisms of resistance. Nanoparticle-based delivery systems are being explored (Rahimi et al., 2019). Therefore, with the current body of evidence, Indolicidin represents a viable candidate for the development of new generation anti-infective agents, due to its ability to provide alternative options for treating chronic, multidrug resistant infection by Mycobacterium species, as such, we believe that it warrants further examination from a structural, mechanistic and translational perspective.

#### Synthetic antimycobacterial peptides

These peptides are either natural peptide derivatives or engineered peptides. Several peptides with anti‐mycobacterial activity show linearity in their actions, with the only exception to plectasin analogous peptide ‘NZX’ (Pruksakorn et al., 2010). These peptides have shown specific activity towards *M. tuberculosis* strains with good *in vivo* stability and promising activity (Yathursan et al., 2019).

#### NZX

The cationic defensin-like peptide NZX has been designed using Plectasin, a fungus-derived antimicrobial protein, which was isolated from *Pseudoplectania nigrella* and shown to be very effective against many types of pathogenic bacteria. The optimized NZX variants (**Table 2**) were then structurally characterized and demonstrated a compact, cysteine-bonded structure (PDB: *6K50*). This structure is consistent with those observed in other fungal defensins and may contribute to the significant stability of NZX in biological systems. NZX was developed for Gram-positive pathogens, and it has came to the forefront of tuberculosis research because of its high level of inhibition against *Mycobacterium tuberculosis* and its ability to show dose-dependent killing effects on both drug-sensitive (H37Rv) and MDR clinical isolates. In mice infected with *M. tuberculosis*, NZX delivered through short-course intratracheal administration resulted in the dramatic reduction of pulmonary CFUs and produced a level of control comparable to that of rifampicin, the most common antibiotic used to treat TB, indicating that NZX may have significant *in vivo* therapy potential. At very high concentrations, NZX is very non-cytotoxic to mammalian macrophage cells. Thus, NZX demonstrates good translational potential and is highly beneficial for all peptides intended for respiratory delivery (Torres et al., 2019). The most significant aspects of datasets on plectasin-derived peptides have been their stability to proteolytic degradation within the body, and as a result, these peptides have the best pharmacodynamic and pharmacokinetic properties when applied against antibiotic-resistant bacteria. Recent findings also suggest that NZX may synergize with other existing antimicrobial agents, thereby decreasing resistance and increasing treatment effectiveness when used together. In addition, characterising the structure of NZX helps to understand how various disulfide linkages and charge distributions contribute to its binding to the exterior of bacterial cells; thus providing valuable information for future peptide engineering efforts (Tenland et al., 2018). Overall, NZX represents a compelling next-generation antimicrobial candidate, structurally robust, highly active against *M. tuberculosis*, including MDR strains, and demonstrably effective *in vivo*, with a safe and very stable profile that supports its advancement toward therapeutic development.

#### GranF2

GranF2 is a synthetic 23-residue antimicrobial peptide in the preclinical stage that mimics the lytic domain of human granulysin. It exhibits potent bactericidal activity against drug-susceptible and MDR *Mycobacterium tuberculosis* by inducing cell wall permeation and osmotic lysis (Andreu et al., 1999). In addition, it also has immunomodulatory properties. GranF2 contains rich patches of lysine and arginine, which provide the peptide a strong positive charge surface that can efficiently interact with the cell membrane. The antimycobacterial activity of this peptide is based on the indirect initiation of apoptosis in mammalian cells, which aids in the removal of the pathogen by the elimination of the intracellular moieties containing the pathogen (Linde et al., 2005; Toro et al., 2006). GranF2 retains the membrane disruption properties of granulysin (Andreu et al., 1999). While not yet in clinical trials, current research focuses on using it as a cell-penetrating carrier to enhance the delivery of other antitubercular drugs into infected macrophages (Ábrahám et al., 2016).Recent updates further advocate for its integration into “smart” nanocarriers to minimize off-target effects and target persistent bacteria (Obaid and Al-Ganahi, 2025). There are currently no approved therapeutic applications for GranF2.

#### Ub2

Cationic peptides (CP) derived from the human lysosomal ubiquitin (Ub) family, such as Ub2 contain a distinct architecture formed by repeated sequences linked together in a long chain and exhibiting a similar folding pattern to that seen in linear polyubiquitin chains. The functional activity demonstrated by Ub-derived CPs shows that they act via similar mechanisms, supporting the theory that CPs derived from ubiquitin can penetrate mycobacterial cells without possessing specific receptor binding sites (Alonso et al., 2007; Foss et al., 2012). Although cytotoxicity of Ub2 are unavailable, several ubiquitin-derived peptides have exhibited low toxicity toward mammalian cells with retaining its broad-spectrum antimicrobial activity (Brogden, 2005).

#### NP-1

Neutrophil peptide-1 (NP-1) is a positively charged antimicrobial peptide that resides in storage granules located in rabbit white blood cells, enabling rapid release from these granules upon detection of foreign microbes. Although it is in the preclinical stage, showing strong inhibition of HSV-1 and HSV-2 entry and spread, making it a candidate topical microbicide (Sinha et al., 2003). The amino acid sequence and 3D structure of NP-1 have not yet been published in any established protein databases; however, researchers have been able to conduct extensive research with respect to the mechanism of action of NP-1 on bacteria. Through the use of NP-1, mycobacterium can be killed through direct interactions with their cell membranes. The mechanism of action of defensin-type peptides, including NP-1, is based on electrical attraction due to the positive charge of the antimicrobial peptide being attracted toward the negative charges present on the outer surface of most bacterial cells (Miyakawa et al., 1996). This interaction with bacterial membranes not only causes a breakdown of the membrane’s stability; it also results in cellular leakage and ultimately leads to a rapid rate of death for these bacteria. One of the benefits of this type of antimicrobial mechanism is that it is unlikely for any strain of bacteria to become resistant to this method. The antimicrobial activity of NP-1 is significant. However, there is limited published data regarding NP-1s cytotoxicity, and there is still a gap in our understanding of the pathway by which NP-1 converts to cytotoxins. Research conducted on neutrophil defensins has shown that these peptides exhibit an optimal ratio of antimicrobial activity versus toxicity to pathogens; thus the antimicrobial activity versus toxicity toward host cells is an example of the significance of NP-1 in nature and that it is produced by the body as a natural defence molecule (Ganz, 2003; Selsted and Ouellette, 2005). In addition, the repeated observations of defensin-mediated disruption of membranes involved with activities against the Mycobacterium species validate the viability of understanding NP-1 as a representative cationic peptide that represents the contributions of leukocytes towards the development of immunity toward antimycobacterial activity and as an initial point for designing new therapeutics based on peptide molecules (Brogden, 2005).

The status (in both clinical and preclinical studies) of the evaluated antimycobacterial peptides varies with respect to the stages of development for each peptide type, as depicted in **Table 3**. The human innate immune peptides, particularly Cathelicidins (LL-37), are currently undergoing Phase 2 clinical trials using human and/or mouse models. However, there is currently no FDA approval for the use of Cathelicidin peptides. Clinical studies conducted with these peptides have primarily investigated their immunomodulatory effects and the efficacy of adjunctive (host-targeted) therapy with two different immunomodulatory compounds (i.e., oral phenylbutyrate and vitamin D3) for the treatment of tuberculosis (TB), using randomized controlled designs. Conversely, Capreomycin (a non-ribosomal bacterial peptide) has been fully approved by the FDA as a second-line treatment for multi-drug resistant tuberculosis (MDR-TB) since 1973 and has comparative clinical utility based on individualized patient data meta-analysis and World Health Organization (WHO) recommendations, as well as established efficacy in guinea pig and mouse pulmonary TB models. Conversely, the synthetic antimycobacterial peptide NZX remains in the pre-clinical phase; however, it does show efficacy in mouse models for tuberculosis and bovine mastitis, but has not yet progressed through phase 1 or phase 2 clinical trials in humans.

**Table 3:** Preclinical and clinical status of the antimycobacterial peptides.

Sl no.		Peptide	Status (preclinical/clinical/approved)	Animal model	References
1.		Human Innate Immune Peptides			
	a)	Cathelicidins/LL-37	Phase 2 clinical trials but no FDA approval.	Mouse and Human	(Rivas-Santiago et al., 2013; Mily et al., 2015; Gupta et al., 2017; Bekele et al., 2018; Rekha et al., 2018; Palacios et al., 2022)
2.		Non-Ribosomal and bacterial peptides			
	b)	Capreomycin	FDA-approved (since 1973) for MDR-TB; second-line therapy: Guinea pig and Mouse pulmonary TB model.	Guinea pig and Mouse	(Nakamura et al., 1964; Garcia-Contreras et al., 2007; Gonzalez-Juarrero et al., 2012; World Health Organization, 2014; Cegielski et al., 2021)
3.		Synthetic Antimycobacterial Peptides			
	c)	NZX	Preclinical; efficacy in TB animal models and bovine mastitis.	Mouse	(Tenland et al., 2018)

## Mechanism of action of AMPs

The antimycobacterial peptides either target the membrane of the bacteria or the biomolecules other than the membrane. Hence, the mechanism of the antimycobacterial peptides can be categorized into.

1. Membrane targeting
2. Non-membrane targeting

### Membrane targeting

**Figure 4:**
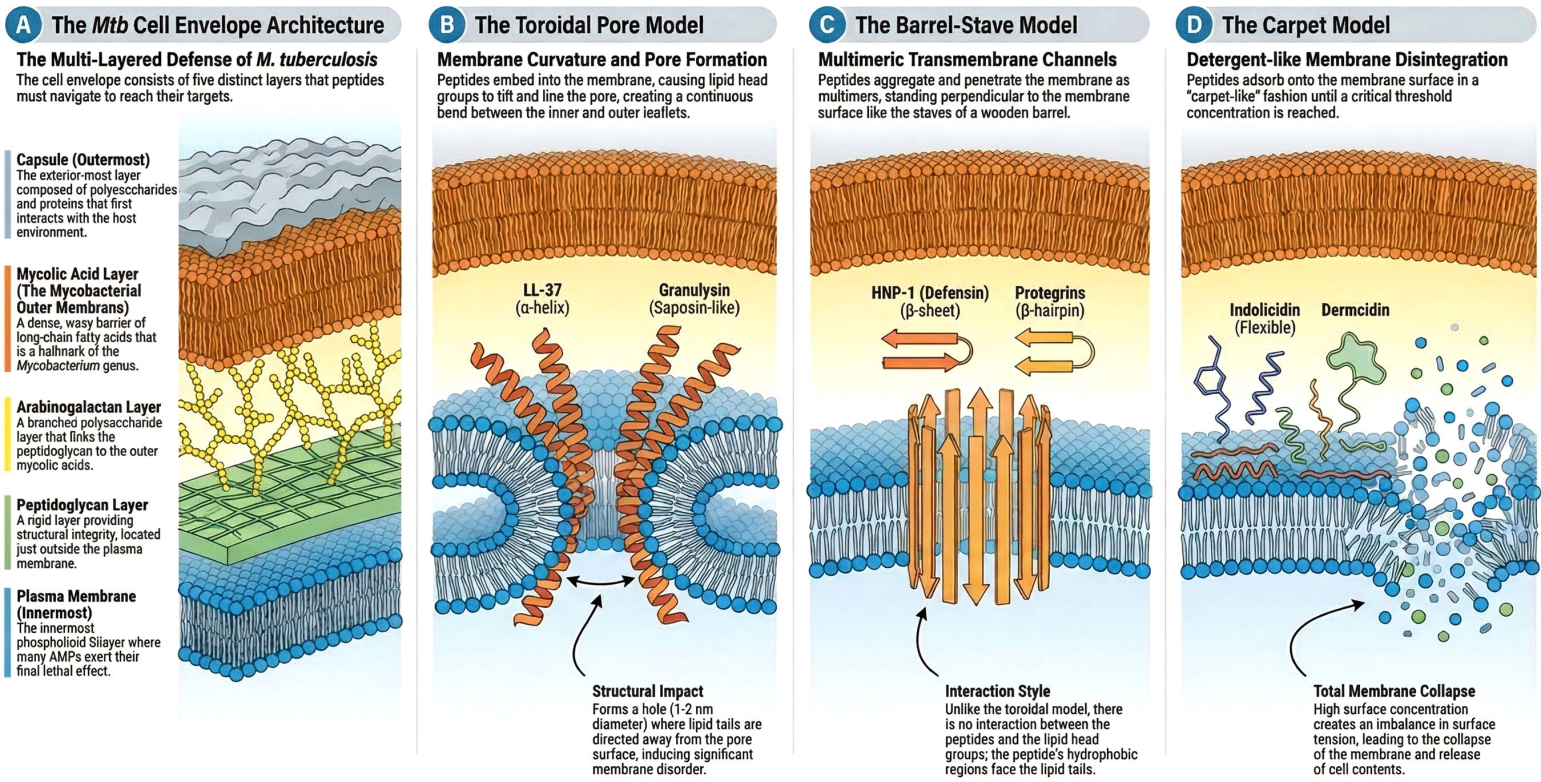
Membrane-targeting mechanisms of AMPs against the Mycobacterium tuberculosis cell envelope. Panel **(A)** on the left details the complex, highly hydrophobic architecture of the M. tuberculosis cell wall, which includes the plasma membrane, a cross-linked peptidoglycan layer, arabinogalactan, and a thick outer layer of mycolic acids. The right panels **(B–D)** illustrate the distinct structural models by which cationic and amphipathic AMPs disrupt this barrier: the toroidal pore model (inducing membrane curvature by hauling lipid heads into the lipid tail), the barrel-stave model (forming transmembrane multimers that cause cytoplasmic outflow), and the carpet model (adsorbing parallel to the membrane, leading to detergent-like disintegration and cell lysis).

Antimicrobial peptides (AMPs) employ a variety of mechanisms to attack the highly complex and formidable cell wall of *Mycobacterium tuberculosis (Mtb).* The Mtb cell wall is characterized by extreme hydrophobicity and low permeability, consisting of a plasma membrane, a cross-linked peptidoglycan layer, arabinogalactan, and a thick outer layer of mycolic acids (Arranz-Trullén et al., 2017; Singh et al., 2018; Maitra et al., 2019). Because most AMPs are small, cationic (positively charged), and amphipathic, they are uniquely suited to interact with the negatively charged surface of the Mtb cell envelope (Torrent et al., 2011; Mehta et al., 2022). They act on the cell wall and membrane through the mechanisms as shown in **Figure 4**.

The most common mechanism by which AMPs act is through direct electrostatic binding to the anionic phospholipids of the bacterial membrane, followed by permeabilization (Lehrer et al., 1993; Méndez-Samperio, 2008). Once they accumulate on the membrane, they disrupt its integrity through specific structural models: Toroidal pore model: AMPs are embedded inside the cell membrane forming a hole with a diameter in the range of 1–2 mm (Matsuzaki et al., 1995; Sengupta et al., 2008; Li et al., 2017). The mode of interaction between the peptide and cell membranes is electrostatic in nature. The toroidal shape pore is formed by the penetration of the AMP, where the lipid heads are pulled into the lipid tail and the tails of the lipids are directed away from the pore on the surface (Mihajlovic and Lazaridis, 2010). This induces a change in the curvature of the membrane and disorderness in the lipid bilayer. The second membrane-targeting model is the Barrel-Stave model. In this model, there is no interaction between the AMPs and head group and the lipid tails (Lohner and Prossnigg, 2009; Bertelsen et al., 2012). In this model, the AMPs aggregate and penetrate as multimers inside the cell membranes. This penetration of the cell membrane leads to the outflow of the cytoplasm.

In some cases, the AMPs can lead to the disintegration of the membrane and cause cell death (Song et al., 2013). The third model is known as the carpet model. In this model, the AMPs are adsorbed on the surface of the membrane in such a way that the polar end faces the solution and the non-polar end faces the membrane bilayer (Matsuzaki et al., 1996). The adsorption of AMPs onto the cell membrane resembles a carpet, and the cell membrane is destroyed in the same way as a detergent. Due to the high surface concentration of the AMPs, there is an imbalance in the charge and surface tension, which leads to the collapse of the cell membrane and the release of the cell contents.

Different types of AMPs exhibit specific modes of action against the Mtb cell wall. Like human neutrophil peptides (HNPs) bind electrostatically to the cell membrane, increasing the permeability of the mycobacterial cell envelope and forming voltage-regulated channels that cause the leakage of intracellular metabolites (Lehrer et al., 1993; Méndez-Samperio, 2008). Granulysins are found to be acting on mycobacterial membranes by stopping the synthesis of liposomes and inducing mammalian cell apoptosis (R and Anbarasu, 2022). Cathelicidins (e.g., LL-37, CRAMP) peptides cause direct membrane disruption and pore formation (Rivas-Santiago et al., 2013). Dermcidin secreted by human sweat glands, inhibits the mycolyl transferase enzyme. This action interferes with the transfer of mycolic acids to the cell wall, severely impairing cell wall synthesis (Banerjee and Gohil, 2016; Niyonsaba et al., 2017). Similarly, Lacticin 3147 inhibits the synthesis of peptidoglycan—a critical structural component of the bacterial cell wall—by binding to the essential peptidoglycan precursor (Martin and Breukink, 2007; Carroll et al., 2010).

In addition to their direct effects on the cell wall, the initial membrane permeabilization allows many AMPs to translocate across the Mtb cell envelope into the cytoplasm. After entering the cell, non-membrane acting peptides interfere with crucial intracellular functions, like binding to genomic DNA (Shu et al., 2019). Many AMPs have the ability to inhibit protease activity, which in turn can inhibit the essential metabolic activities of the pathogen (Anbarasu and Proteins, 2023). In addition, some work by inhibiting replication and transcription, or by inhibiting vital enzymes like ATPases and DNA topoisomerase I which helps in the formation of the DNA (He et al., 2017).

### Non-membrane targeting

**Figure 5:**
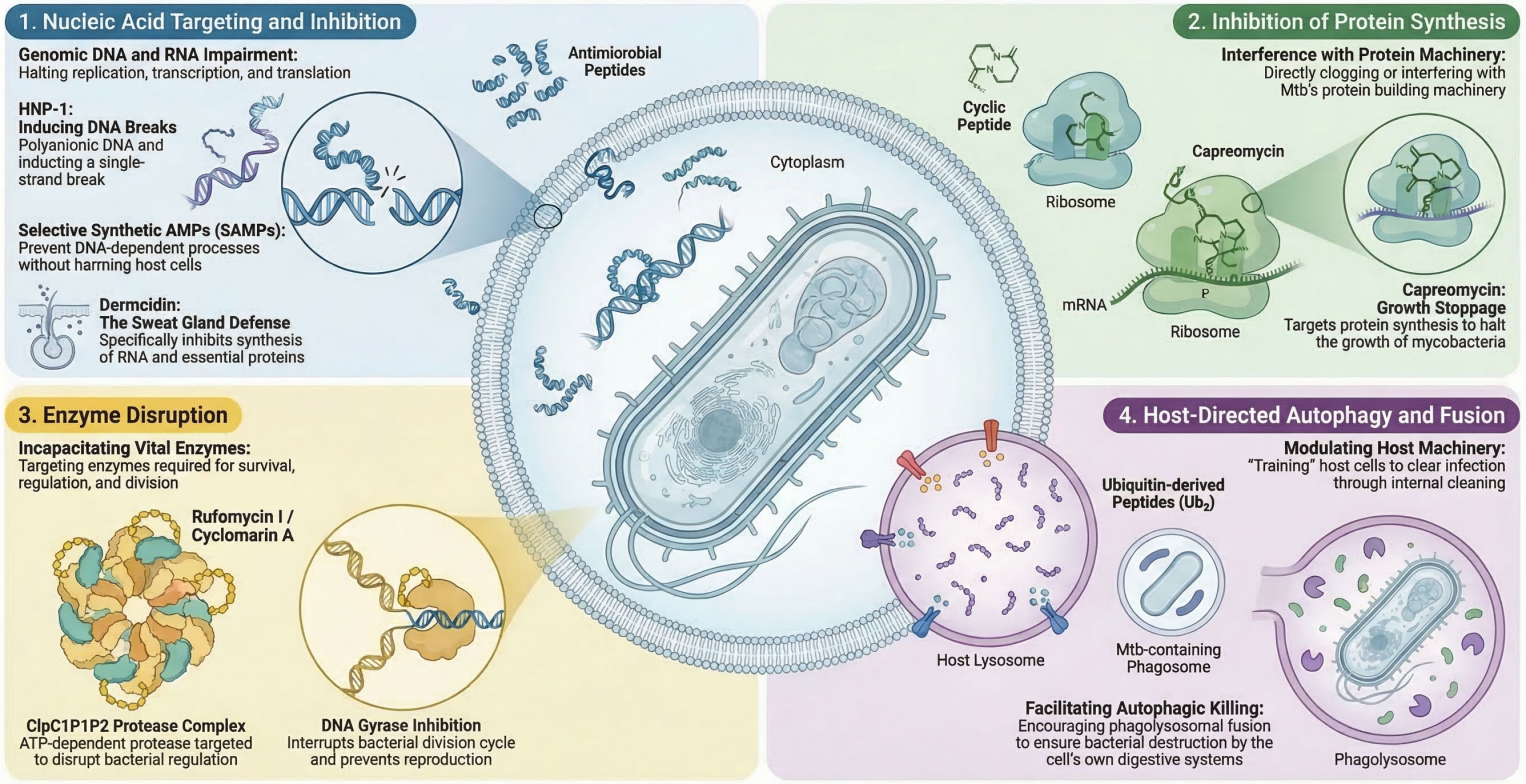
Intracellular and non-membrane targeting mechanisms of antimycobacterial peptides. This image outlines how specific AMPs translocate across the bacterial cell envelope to neutralize *M. tuberculosis* without primarily relying on immediate membrane lysis. Depicted mechanisms include binding to genomic polyanionic DNA to inhibit replication and transcription, directly interfering with the protein biosynthesis machinery and molecular chaperones, and incapacitating vital bacterial enzymes (such as the ClpC1P1P2 protease complex). Additionally, it illustrates how some host-derived peptides, such as ubiquitin-derived AMPs, indirectly promote bacterial clearance by inducing host-directed autophagy and facilitating phagolysosomal fusion within infected macrophages.

AMPs can also exert their antimicrobial function by inhibiting protein biosynthesis, protease activity, or disruption of nucleic acids as shown in **Figure 5**. The inhibition of protein biosynthesis is mainly due to the interference caused by AMPs with the enzymes involved in molecular chaperones to induce proper folding (Matsuzaki et al., 1996).This affects the transcription and translation process of the proteins.

In cases of nucleic acid targeting and inhibition process certain AMPs are able to cross the mycobacterial lipid bilayer, migrate into the cytoplasm, and bind directly to intracellular nucleic acids. This severely affects the ability for the replication and transcription of genetic material in bacteria. This interaction is driven by the electrostatic attraction between the polyanionic backbone of bacterial DNA or RNA and the positively charged amino acid residues of the peptides (Giuliani et al., 2007; Le et al., 2017). We found that HNP-1 binds to polyanionic molecules like genomic DNA, inducing single-strand DNA breaks and inhibiting DNA biosynthesis (Gera and Lichtenstein, 1991; Sharma and Khuller, 2001). Similarly, synthetic antimicrobial peptides are uniquely selective; they can enter Mtb without being toxic to the host’s cells and bind to genomic DNA, preventing DNA-dependent processes and leading to bacterial death (Sharma et al., 2015). Dermcidin (secreted by human sweat glands) inhibits RNA and protein synthesis (Li et al., 2009; Banerjee and Gohil, 2016).

The second mode of non-membrane targeting is the inhibition of protein biosynthesis. Several cyclic peptides directly interfere with the Mtb protein synthesis machinery. They act upon bacterial translation machinery by shutting it down. These peptides directly interact with mycobacterial ribosomes to prevent the synthesis of proteins necessary for bacterial survival, virulence, and replication (Carnero Canales et al., 2026). Capreomycin, which is produced by Streptomyces species, specifically inhibits protein synthesis to stop mycobacterial growth (Shiba et al., 1976; Akbergenov et al., 2011).

The third mode of targeting is aimed at enzyme disruption. AMPs can target and incapacitate vital bacterial enzymes required for Mtb survival, protein regulation, and replication. They mostly target the highly conserved bacterial protein degradation machinery, like the caseinolytic protease complex (ClpP1P2) and respective regulatory ATPases, such as ClpC1 which functions to maintain proteostasis by degrading misfolded or toxic proteins (Yang et al., 2023). ClpC1P1P2 protease complex is an ATP-dependent protease uniquely targeted by specific cyclic peptides such as rufomycin I, and cyclomarin A (Schmitt et al., 2011; Choules et al., 2019).

Finally, some AMPs initiate host-directed autophagy and phagolysosomal fusion. Rather than attacking the bacteria’s internal structures directly, they inflict non-membrane effects by modulating the host’s intracellular defense mechanisms to clear the infection. Ubiquitin-derived peptides (Ub2) located within the host’s lysosomes promote the fusion of the Mtb-containing phagosome with the lysosome, facilitating autophagic killing of the bacteria (Foss et al., 2012).

## Translational roadmap and prioritization for clinical development

As we have previously discussed the properties of AMyPs and their clinical significance, we have ranked them on three key translational metrics: MDR/XDR activity, *in vivo* efficacy, and delivery feasibility (**Table 4**).

**Table 4:** Tiered ranking of all antimycobacterial peptides.

Priority level	Criteria	Peptides
Tier 1: High Translational Priority	MDR-active, *in vivo* efficacy demonstrated, feasible delivery	NZX: A synthetic, highly stable peptide that exhibits dose-dependent killing against both drug-sensitive and multidrug-resistant (MDR) clinical isolates. It provides robust *in vivo* efficacy comparable to rifampicin and is highly feasible for respiratory delivery. Cyclomarin A: A cyclic non-ribosomal peptide that effectively kills multidrug-resistant strains by targeting the ClpC1 protease. Its rigid macrocyclic structure provides exceptional antiproteolytic stability and high feasibility for enteric/intracellular delivery. Rufomycin I (Ilamycin A): A cyclic non-ribosomal peptide highly potent against MDR and XDR strains via ClpC1 inhibition. It is stable in aqueous solutions and successfully penetrates cells. Capreomycin: An FDA-approved cyclic peptide antibiotic utilized as a second-line therapy for MDR-TB. It is an extremely potent ClpC1 inhibitor with established clinical delivery.
Tier 2: Moderate Priority - Require Development	Excellent antimycobacterial activity but stability/delivery/toxicity limitations	LL-37: Demonstrates excellent intracellular activity and immunomodulation, but its short *in vivo* half-life and susceptibility to proteolytic degradation hinder systemic translation. Granulysin: Offers potent cytolytic activity, but as a host-derived peptide, it requires integration into advanced nanocarrier systems to remain stable and bypass degradation. Protegrins (e.g., PG-1): Show strong membrane-disrupting activity against MDR strains, but face major clinical limitations due to toxicity concerns (analogs like Iseganan failed Phase 3 trials). Indolicidin: Exhibits broad antimycobacterial activity, but clinical development is currently limited by hemotoxicity and protease degradation; relies heavily on nanoparticle delivery exploration. Lacticin 3147: While its potency against the bacterial cell wall can exceed rifampicin, it requires complex solid lipid nanoparticle or hydrogel delivery systems to function therapeutically. GranF2: A potent synthetic peptide against MDR strains, but necessitates integration into “smart” nanocarriers to minimize off-target effects and target persistent bacteria. Dermcidin: Has good broad-spectrum effects by inhibiting cell wall synthesis, but is currently in preclinical stages requiring development for specific systemic therapies. HNP-1 & HNP-2: Possess strong bactericidal capabilities, but their translation is restricted by high production costs, potential biofilm-promoting effects (HNP-1), and the need for novel recombinant delivery mechanisms. HBD-3 & HBD-4: Effective in direct killing and immune modulation, but require the development of protease-resistant analogs and biomaterial-based delivery systems to be viable. NP-1: Demonstrates rapid membrane disruption, but currently lacks comprehensive toxicity data toward host cells, limiting its use mostly to a candidate topical microbicide.
Tier 3: Lower Priority - Indirect Mechanisms	Supportive role, indirect antimycobacterial effects, or biomarker applications	Lactoferrin: Acts indirectly by sequestering essential metals (iron) and regulating mucosal immunity rather than directly lysing the pathogen. Hepcidin: Functions primarily by limiting the release of recycled iron from macrophages, starving the intracellular bacteria of nutrients. Azurocidin: Acts primarily as an adjunct, host-directed therapy, and predictive biomarker for sepsis rather than a standalone direct therapeutic. Elastases: Indirectly assist in bacterial clearance by remodeling host tissues, forming Neutrophil Extracellular Traps (NETs), and immunomodulation. Ub2: Exerts indirect antimycobacterial effects by inducing host-directed autophagy and promoting phagolysosomal fusion. HNP-3: Demonstrates the lowest microbicidal potency among defensins and is instead being explored as a diagnostic biomarker and vaccine adjuvant. HBD-1: While it acts as a primary barrier, recent research has pivoted heavily toward its use as a diagnostic and prognostic biomarker for TB.

### Synergistic combination strategies with current TB regimens

AMPs and AMP-like molecules provide a potent method for improving existing and novel anti-tuberculosis treatments such as BPaL and BPaLM. By actively disrupting the highly impermeable mycobacterial cell membrane, AMPs neutralize bacterial efflux pumps, facilitate massive intracellular accumulation of antibiotics, reduce the required therapeutic doses, and delay the emergence of resistance (Sharma et al., 2001). The combined application of AMPs with conventional frontline drugs like Isoniazid, Rifampicin, and Ethambutol has been observed to produce a synergistic outcome in both *in vitro* and *in vivo* experiments. The combination of Isoniazid (INH), Rifampicin (RIF), and Human Neutrophil Peptide-1 (HNP-1) has been observed to decrease the minimum inhibitory concentrations (MIC) by eightfold, leading to a substantial improvement in clearing pathogens from the lungs, liver, and spleen in infected individuals (Sharma and Khuller, 2001; Sharma et al., 2001; Kalita et al., 2004; Roque-Borda et al., 2025). Similarly, both Protegrin-1 (PG-1) and HBD-1 significantly reduce the *in vitro* growth of drug-susceptible and MDR Mtb strains when combined with INH, proving much more effective than using the peptide or the antibiotic alone (Fattorini et al., 2004). Several synthetic peptides, like granulysin-derived peptide (GranF2) have shown strong synergy with Ethambutol (EMB) by increasing cell-wall permeation, effectively clearing intracellular Mtb from macrophages without harming host cells (Martineau et al., 2007). Whereas NZX and its variant NZ2114 exhibit synergistic and additive effects when combined with EMB or INH, successfully lowering bacterial loads *in vivo* against murine TB (Rao et al., 2021; Davids et al., 2025). One group tried encapsulating host defense peptides like K4, Ub2, and Aurein-1.2 with Isoniazid (INH) in PLGA microspheres and this approach led to the better perforation of the bacterial cell membrane, accelerating the permeation and dose efficacy of INH (Sharma et al., 2019).

BPaL (bedaquiline, pretomanid, linezolid) and BPaLM (BPaL + moxifloxacin) regimens are highly effective and globally recommended for MDR-TB and XDR-TB (Gualano et al., 2024; Apgar and Suryani, 2025). Recent research has identified targeted synergistic relationships between AMPs and the specific components of these newer regimens. There are reports on pairing AMPs with moxifloxacin against Mtb. Host defense peptides like LL-37 and human β-defensin 3 (HBD3) have demonstrated powerful antimicrobial synergy with moxifloxacin against bacterial infections, establishing the broad synergistic potential of combining membrane-active peptides with the fluoroquinolone component of the BPaLM regimen (Nuding et al., 2014).

### Translational challenges and limitations

However, till yet the clinical translation of novel TB therapeutics, particularly AMPs and host-directed therapies, is still facing several critical gaps across biological, pharmacological, and regulatory domains. A primary barrier to the clinical use of AMPs is their high susceptibility to proteolytic degradation by both host enzymes (in the digestive tract and blood) and bacterial proteases (Haney and Hancock, 2013; Roque-Borda et al., 2025). This vulnerability, combined with rapid hepatic and renal clearance, results in a very short *in vivo* half-life and poor systemic stability (Mahlapuu et al., 2016; Rezende et al., 2021). AMPs, unlike traditional drug-likeness rules, tend to limit oral bioavailability and restrict absorption and distribution profiles (Azman et al., 2022; Talha and Roque-Borda, 2026). Many AMPs exhibit a narrow therapeutic window due to cytotoxicity toward mammalian host cells. Because of their membrane-disrupting mechanisms, high hydrophobicity, and cationicity, they can cause significant hemolysis of erythrocytes and damage to epithelial cells at therapeutic concentrations (Zharkova et al., 2019; Mahlapuu et al., 2020; Rai et al., 2022a, 2022b; Gupta et al., 2025). Additionally, non-specific immunomodulatory effects risk triggering excessive, harmful inflammation, and there is a lack of comprehensive, long-term toxicological safety data for humans (Saha et al., 2024). There also exists the formidable physical barrier – tuberculous granuloma. The heterogeneous microenvironment of a granuloma—characterized by dense fibrous tissue, necrotic caseum, hypoxia, and acidic zones—severely limits the penetration and even distribution of peptides and nanocarriers, making it highly difficult to reach the dormant or intracellular Mtb residing within host macrophages (Fang et al., 2024; Carnero Canales et al., 2026). In addition, *in vitro* assays fail to fully replicate the complex dynamics of a bacterial infection within a human host, such as pharmacodynamics, host immune responses, and the intricate tissue microenvironments (Morales-Durán et al., 2024; Roque-Borda et al., 2025). Consequently, many candidates that show excellent efficacy in the lab fail in clinical trials (Morales-Durán et al., 2024; Roque-Borda et al., 2025). Furthermore, clinical data evaluating the long-term impacts of endogenous AMP induction or host-directed therapies remains grossly insufficient (Arranz-Trullén et al., 2017; Kolloli and Subbian, 2017). There is also present manufacturing, economic, and regulatory obstacles. Given that TB predominantly afflicts low- and middle-income countries with constrained healthcare budgets, the high cost of chemical synthesis and complex purification processes is a major obstacle to the large-scale production of AMPs (Magana et al., 2020; Fang et al., 2024; Luo et al., 2024; Roque-Borda et al., 2025). Though AMPs are generally less prone to resistance than conventional antibiotics due to their multifaceted modes of action, the risk still remains. Under the prolonged selective pressure required for TB treatment, microorganisms may evolve adaptive resistance mechanisms against AMPs (Arranz-Trullén et al., 2017; Talha and Roque-Borda, 2026).

### Future directions

To overcome gaps like rapid proteolytic degradation, short half-life, and poor systemic bioavailability of AMPs, researchers are adapting sophisticated nanocarrier platforms. The AMPs are being encapsulated in liposomes, solid lipid nanoparticles, and polymers like poly(lactic-co-glycolic acid) (PLGA), chitosan, and hyaluronic acid (Saini et al., 2025; Carnero Canales et al., 2026) or inorganic platforms, such as mesoporous silica nanoparticles (MSNs), which offer high peptide loading capacity (Carnero Canales et al., 2026). These nanocarriers protect the peptides from enzymatic degradation and facilitate targeted uptake by alveolar macrophages via specific receptors like CD44 (Silva et al., 2016b). Some AMPs are delivered via dry powder inhalers or aerosols that allow for localized, direct delivery to the deep lungs. This approach maximizes drug concentration at the primary site of *Mycobacterium tuberculosis* (Mtb) infection while minimizing systemic toxicity and side effects (Cunha et al., 2019; Shao et al., 2023, 2024). The discovery and optimization of anti-TB peptides have been vastly accelerated by computational methods such as machine learning (ML) and artificial intelligence (AI). AI algorithms process massive datasets from dedicated AMP repositories (like AntiTbPdb, DRAMP, and DBAASP) to predict sequence-to-function relationships. These tools optimize antimicrobial potency, enhance stability, and minimize host cytotoxicity prior to physical synthesis (Carnero Canales et al., 2026). The predictive computational techniques like molecular docking and molecular dynamics (MD) simulations help in the prediction of peptide binding sites and evaluate the flexibility, stability, and membrane-penetrating behavior of AMPs in dynamic, simulated physiological environments (Roque-Borda et al., 2025). The chemical stabilization modifications, such as cyclization, PEGylation, and the incorporation of non-natural D-amino acids, are employed to shield the peptides from host proteases and extend their *in vivo* lifespan (Roque-Borda et al., 2025). There are also targeted engineered molecules called specifically targeted AMPs (STAMPs). They feature two domains, one of which binds specifically to the pathogen and the other destroys it. STAMPs are observed to eradicate the infection without damaging host mammalian cells or beneficial commensal microbiota (Saini et al., 2025; Talha and Roque-Borda, 2026). And lastly, to combat the manufacturing and scalability of production of AMPs, recombinant DNA technology is being used. Scalable production platforms using microbes (e.g., *E. coli, Pichia pastoris*), transgenic plants, and fungi are selected for cost-effective manufacturing alternatives (Yadav et al., 2024; Hong et al., 2025; Liu et al., 2025). The future directive of AMPs is now leaning toward synergistic combination therapies as the primary clinical goal to integrate AMPs with conventional first-line antibiotics (like rifampicin and isoniazid) or next-generation drugs (bedaquiline and proteomanid). Another major future priority is to successfully bridge the translation of *in vitro* and *in vivo* findings into clinical settings.

## Conclusion

Antimycobacterial peptides (AMPs) are a novel addition to the anti-tuberculosis therapies used for the treatment of patients with tuberculosis. As this disease progresses and patients develop more resistance to traditional therapy, AMPs have shown significant promise in treating this infection. This review includes evidence that AMPs can effectively kill *Mycobacterium tuberculosis*, using multiple mechanisms of action including: 1) damage to or disruption of the mycobacterial cell membrane, 2) interference with the cell’s intra-cellular targets, 3) blockade of key mechanisms needed to maintain the integrity of the cell (proteostasis), and 4) alteration of human host immune responses. Because AMPs are effective against both actively multiplying and latency populations of *M. tuberculosis*, they have the potential to significantly shorten the overall duration of treatment and improve patient outcomes. The development of AMPs as a treatment modality for tuberculosis should focus on maximising their *in vivo* stability and efficacy through improved delivery and manufacturing systems, as well as the development and use of more affordable host-directed combination therapies for use with the currently available first-line anti-TB drugs. Ultimately, AMPs represent an excellent opportunity to develop new tools to treat drug-resistant tuberculosis and improve the way we develop and provide care to TB patients worldwide.
